# Botanical Report

**Published:** 1811-08

**Authors:** 


### BOTANICAL REPORT.

The physiology of vegetables has been but little prosecuted in this country of late years, considering its great importance, though Dr. Grew in the 17th century laid a valuable foundation for this study. The French botanists have pursued the subject with more ardour. It has not been however by any means totally neglected here. Mr. Knight in particular has applied himself with great assiduity to this subject, and, in various papers published in the Philosophical Transactions, has, we think, thrown more light upon the theory of vegetation than any of his predecessors, at home or abroad. Nor should the labours of Mrs. Ibbetson in this line be passed unnoticed. This ingenious lady has made a number of very interesting observations and experiments, which certainly throw light upon the subject, but the very high magnifying power she uses, aided by the warmth of her imagination, seems often to have led her into the regions of fancy; and the little knowledge she has of what has been already done, and even of the terms used by preceding writers, throws an obscurity over her writings, which makes it very difficult to understand them.

Mr. Knight's opinions and observations, though highly luminous and satisfactory with regard to the immediate subject of this inquiry, yet being written at different times, and with a particular view to the illustration sometimes of one point and sometimes another, are not easily connected together, so as to form in the mind a clear idea of his theory. We suppose that this difficulty has been felt by many, as well as ourselves; and some of his friends have urged him to give a connected view of his theory of vegetation, which he has done in a very satisfactory manner in a paper upon the culture culture of the Melon, in the Transactions of the Horticultural Society, pnblished at page 217 of their first volume. Of this theory, as there delivered, we shall attempt to give a concise view, nearly in the words of the author.

"In the organs of the seed, but principally in the cotyledons, as much of the concrete sap of the parent plant is lodged as is sufficient to feed its offspring, till that has attached itself to the soil, and become capable of absorbing and assimilating new matter. The organizable matter probably exists in the cotyledons of the seeds, in the same state as it exists in the alburnum of trees; and, like that, it apparently undergoes considerable changes before it becomes the true circulating fluid of the plant. In some it becomes saccharine, in others acrid and bitter during germination. In this process the vital fluid is drawn by the caudex of the plumule, or bud, through vessels which correspond with those of the bark of the future tree; and are indeed cortical vessels.

"From the point of the caudex (erroneously called the *radicle**) springs the first root, which is, at this period, without alburnum; and, if uninterrupted by obstacles in its way, constantly descends in a straight line towards the centre of the earth, in whatever situation the seed may happen to be placed.

Soon after the first root has been emitted, the caudex elongates, and takes a direction directly opposite to that of the root; and in many plants, raises the cotyledons out of the ground, which then become the seed-leaves of the plant. During this period the young plant derives its nutriment almost always from the cotyledons or seed-leaves; and if those are destroyed, it perishes.

"The bark of the root now begins to deposit alburminous or woody matter; and, as soon as it is formed, the sap, which had hitherto only descended through the cortical vessels, begins to ascend through the alburnum. The plumule in consequence elongates, its leaves enlarge and unfold; and a set of vessels, which did not exist in the root, are now brought into action. These, which I have called the central vessels, surround the medulla, and, between it and the bark, form a circle upon which the alburnum is deposited by the bark, in the form of wedges, or like the stones of an arch. Through these vessels, which diverge into the leaf-stalks, the sap ascends, and is dispersed through the vessels and parenchymatous substance of the leaf. And in this organ, the fluid, recently absorbed from the soil, becomes converted into the true sap or blood of the plant. And, as this fluid, during germination, descended from the cotyledons and seed leaves of the plant, so it now descends from its proper leaves, and adds, in its descent, to the bulk of the stem and the growth of the root. Alburnum is also deposited in the stem of the plant below the proper leaves, as it was previously deposited below the seed-leaves. And from this spring other cen-

* It was this term of *radicle* which misled Mrs. Ibbetson, who understood by it the *root*; and is surprised that botanists should speak of a part, as existing in the embryo of the seeds, which she declares never can exist prior to germination.

tral vessels, which give existence to, and feed, other leaves and buds.

"A portion of the true sap appears, in its descent down the bark, to secrete into the alburnum, through passages correspondent to the anastomosing vessels of the animal economy. Hence the ascending fluid becomes mixed with a portion of the descending sap in the alburnum.

"The full-grown leaves prepare the fluid which generates other young leaves, the health and growth of which are as much dependant upon the full-grown leaves, as those, when first formed, were upon the cotyledons.

"The power of each proper leaf to generate sap, in any given species and variety of plant, appears to be in a compound ratio of its width, its thickness, aud the exposure of its upper surface to the light in a proper temperature. The mature leaves increase rapidly in proportion to the young leaves, and the creation of sap consequently exceeds the expenditure. It is therefore accumulated during a succession of weeks, or months, or years, according to the natural habits and duration of the plant; and varying considerably according to the soil and climate. The sap, thus generated, is deposited in the bulb of the tulip, in the tuber of the potatoe, in the fibrous roots of grasses, and in the alburnum of trees, during winter; and is dispersed through their foliage and bark during the spring and summer.

"When the plant has attained puberty, a portion of its sap is expended on its blossoms and fruit, which are fed by vessels apparently similar to those of the succulent, annual shoot and leaf-stalk, and which probably convey a similar fluid; for a bunch of grapes grew and ripened, when grafted on a leaf-stalk.

The fruit or seed-vessel appears to be generated always by the prepared sap of the plant, and its chief office to be to adapt the fluids to the proper nourishment of the seed.

Mr. Knight has illustrated the above theory by an application of it to the culture of the melon; a fruit which is often found to be so defective in richness and flavour, as to be hardly worth cultivating. This defect Mr. Knight found by experiment to be owing to the want of a sufficient number of leaves, exposing their upper surfaces to the light. For the stems and foot-stalks of the melon under the hot-bed frame are so weak, that when the leaves are displaced from their proper position, they are not able to regain it. This observation led him to direct that more care should be taken to preserve the leaves in their natural position, with the upper surfaces exposed to the light, which was effected by the aid of little wooden hooks, with which the trailing stems, and even the footstalks of the leaves were secured in their proper places; and by avoiding pouring the water in the usual way upon the leaves of the plant; using instead of a common watering pot, one with a spout adapted for pouring the water upon the tiles which cover the bed without touching the leaves. By this management Mr. Knight found that his melons were no longer defective in richness and flavour.

It may be of use to mention here, that the variety of melon which Mr. Knight exclusively cultivates, on account of its superior flavour, and which we believe is little known to cultivators in general, is the one that was imported by Mr. J. Hawkins from Salonica. The form of this variety is nearly spherical, without any depressions upon its surface. It is of a golden colour, and its flesh perfectly white. This kind, Mr. Knight says, continues to improve in flavour and richness till it becomes externally soft, and betrays some symptoms of decay. The consistence of its flesh is then nearly that of a water melon; and its taste so sweet, that few will think it improved by the addition of sugar. The weight of a good melon of this variety is about seven pounds.

The tenth volume of the Transactions of the Linnæan Society is published.

Dr. Smith has given us a translation of Linnæus's Tour in Lapland, now first published from the manuscript journal. It is in two volumes octavo, and is illustrated by wooden cuts, being fac-similes of the pen and ink sketches in the original.

A journal of a (botanical) Tour in Iceland, by Mr. William Jackson Hooker, is printed, but not published.

The first volume of the Transactions of the Wernerian Society, Edinburgh, is only interesting to the botanist, on account of a paper on the natural order of *Contortæ* of Linnæus, by Mr. Brown, of which we hope at a future time to give some account to our readers.

